# VEXAS syndrome: a comprehensive clinicopathologic and genetic analysis of a predominantly Indian cohort

**DOI:** 10.1007/s12308-026-00695-5

**Published:** 2026-05-16

**Authors:** Dheeraj Chinnam, Aadya Kerkar, Rohit Gulati, Prateek Bhatia, Sreejesh Sreedharanunni, Praveen Sharma, Minu Singh, Shikha Shah, Narayanan Baskaran, Ramya Puligari, Neha Avtarkrishan Ganju, Prakashini Mruthyunjaya, Lekshmon K.S., Anuradha Bishnoi, Dipanker De, Sarath Chandra Mouli Veeravalli, Vishvdeep Khushoo, Avtar Krishan Ganju, Prasanta Padhan, Paul Antony, Vikas Gupta, Anand Balakrishnan, Arushi Sharma, Dikshat Gopal Gupta, Aman Sharma, Pankaj Malhotra

**Affiliations:** 1 Present Address: Department of Pathology and Laboratory Medicine, Indiana University School of Medicine, Indianapolis, IN USA; 2 Department of Pathology, Postgraduate Institute of Medical Education and Research, Chandigarh, India; 3 Clinical Pathology & Laboratory Medicine, Indiana University School of Medicine, Indianapolis, IN USA; 4 Department of Pediatrics (Hematology & Oncology Division), Postgraduate Institute of Medical Education and Research, Chandigarh, India; 5 Department of Hematology, Postgraduate Institute of Medical Education and Research, Chandigarh, 160012 India; 6 Department of Dermatology, Venereology and Leprosy, Postgraduate Institute of Medical Education and Research, Chandigarh, India; 7 Department of Rheumatology and Clinical Immunology, Krishna Institute of Medical Sciences, Secunderabad, Telangana India; 8 Ganju Hematology Clinic, Nagpur, Maharashtra India; 9 Department of Clinical Immunology and Rheumatology, Kalinga Institute of Medical Sciences, KIIT University, Bhubaneswar, Odisha India; 10 Department of Clinical Hematology and Oncology, Postgraduate Institute of Medical Education and Research, Chandigarh, India; 11 Department of Clinical Hematology and Rheumatology, Amala Institute of Medical Sciences, Kerala, India; 12 Rheumatology, Fortis Hospital, Mall Road, Ludhiana, Punjab India; 13 Department of Urology & Pathology, The Robert H. Lurie Comprehensive Cancer Center, Northwestern University Feinberg School of Medicine, Chicago, IL 60611 USA; 14 Department of Internal Medicine (Rheumatology and Immunology Division), Postgraduate Institute of Medical Education and Research, Chandigarh, India

**Keywords:** VEXAS syndrome, UBA1 mutation, Autoinflammatory diseases, Myelodysplastic syndromes, Cytoplasmic vacuoles, Somatic mutation, Neutrophilic dermatosis

## Abstract

**Background:**

VEXAS syndrome is a recently recognized, acquired monogenic adult onset hemato-inflammatory syndrome characterized by somatic mutations within the *UBA1* gene. The acronym VEXAS stands for vacuoles, E1 enzyme, X-linked inheritance, autoinflammatory tendencies, and somatic mutations. It presents as a severe progressive disease displaying varied characteristics that bridge hematologic and rheumatologic domains. Herein, we describe a series with a detailed evaluation of 11 cases of VEXAS syndrome.

**Materials and methods:**

A comprehensive retrospective analysis of patients diagnosed with VEXAS syndrome over the last 5 years (2020–2025) was conducted. Data on clinical presentation, histopathological findings, genetic characteristics, and outcomes were recorded for systematic characterization.

**Results:**

A total of 11 cases of VEXAS syndrome were identified. All the patients were males with an age range from 42 to 77 years. Prominent clinical characteristics included history of fever (11), arthritis/arthralgia (10), inflammatory skin lesions (7), vasculitis (6), ocular inflammatory conditions (6), relapsing polychondritis (6), unprovoked venous thrombosis (5), and auricular chondritis (4). Persistent unexplained cytopenia was present in all the patients, manifesting as anemia (10, 8 of which were macrocytic), thrombocytopenia (4), and neutropenia (2). Bone marrow examination was performed in nine cases, five showed morphologic dysplasia. Furthermore, all nine cases characteristically showed cytoplasmic vacuolations in hematopoietic precursors. UBA1 somatic mutations included p.Met41Thr (c.122 T>C Exon 3) (55%), p.Met41Val (c.121 A>G Exon 3) (27%), p.Met41Leu (c.121 A>C Exon 3) (9%), and a variant in the acceptor splice site of Exon 3 (c.118 G>C) (9%). p.Met41Val is associated with inferior overall survival (OS).

**Conclusion:**

This study characterizes the clinical, morphologic, and laboratory features of VEXAS syndrome and presents the first comprehensive patient cohort from India. With a complex and heterogeneous clinical profile, awareness of the disease is particularly essential among hematologists, rheumatologists, and dermatologists for accurate diagnosis and management.

## Introduction

VEXAS syndrome (vacuoles, E1 enzyme, X-linked inheritance, autoinflammatory tendencies, and somatic mutations) is a recently recognized, acquired monogenic adult-onset hemato-inflammatory syndrome marked by somatic mutations within the UBA1 gene, which encodes the ubiquitin-activating enzyme E1 (1,2). First described in 2020 in a study involving 25 adult males, VEXAS syndrome was shown to present with adult-onset inflammatory conditions along with myeloid dysplasia [1]. Genetic sequencing in these cases facilitated the detection of previously unknown acquired somatic mutations in the* UBA1* gene [3]. *UBA1* encodes the key E1 enzyme that triggers protein ubiquitination in the cell cytoplasm. *UBA1* has two isoforms: UBA1a, the nuclear isoform consisting of 1058 amino acids, and UBA1b, the cytoplasmic isoform comprising 1018 amino acids due to translation initiation at position 41 methionine (p.Met41). Most documented pathogenic mutations associated with VEXAS syndrome involve Exon3, which leads to amino acid substitutions at the locus p.Met41. Approximately 50% of reported VEXAS cases are associated with a specific mutation, c.122T>C (p.Met41Thr), which substitutes methionine with threonine. Another 21% of cases are attributed to the mutations c.121A>G and c.121A>C, leading to the substitutions p.Met41Val and p.Met41Leu, respectively. These mutations disrupt the initiation of the cytoplasmic isoform UBA1b, resulting in its loss of expression. Consequently, senescent and misfolded proteins are not ubiquitinated and degraded by the proteasome, leading to their intracellular accumulation. This accumulation acts as a danger signal, activating the innate immune system and triggering an autoinflammatory response, contributing to systemic inflammation and tissue damage [4–8]. Mutations within UBA1 are confined to the myeloid lineage due to selective clonal expansion bias in myeloid compartment driven by UBA1 mutation, combined with negative selection against mutant lymphoid cells. Due to its location on the X chromosome, the disease primarily affects males. In women, mutation in one copy often results in milder symptoms or the absence of the disease. Since its initial recognition, many cases of VEXAS have been diagnosed, highlighting a higher prevalence than initially perceived [2, 9].

VEXAS is a severe, progressive disease with overlapping features spanning the rheumatologic, hematologic, and dermatologic domains. Patients frequently present with constitutional symptoms such as relapsing fever, malaise, and myalgia, along with an array of inflammatory manifestations involving the skin, cartilage, joints, respiratory system, and blood vessels [3, 10, 11]. Hematologically, cytopenias such as macrocytic anemia and thrombocytopenia are common in cases of VEXAS and may progress into myelodysplastic syndrome (MDS)[12]. Additionally, plasma cell dyscrasias and unprovoked venous thromboembolisms have been reported later in the disease course [13]. Conventionally, VEXAS syndrome has been managed with glucocorticoids and traditional disease-modifying antirheumatic drugs (DMARDs) [2, 14]. However, most often the disease is refractory to treatment and runs a fatal course.

Despite growing awareness, the diagnosis remains challenging due to its heterogeneous clinical presentation, overlap with other rheumatologic and hematologic disorders, and limited availability of targeted molecular testing for *UBA1* mutations. Management is challenging, with no targeted treatment algorithms and variable responses to immunomodulatory therapies. This study emphasizes the need for heightened clinical suspicion, especially in patients with refractory and unexplained inflammation and cytopenia's and aims to provide a detailed characterization of the clinical, pathological, and genetic features of VEXAS, as well as associated treatment outcomes, to aid in early recognition and effective management of this emerging disease entity.

## Materials and methods

This study included eleven patients diagnosed with VEXAS syndrome between December 2020 and May 2025. Nine patients were evaluated at the Postgraduate Institute of Medical Education and Research (PGIMER), Chandigarh, India, and two at Indiana University School of Medicine (IUSM), USA.

Comprehensive clinical, laboratory, therapeutic, and outcome data were collected using standardized case history forms and institutional electronic medical records.

Genetic analysis for the PGIMER included targeted genetic testing via amplification of the *UBA1* gene region encompassing exons 2 and 3 along with the intervening intronic sequences using polymerase chain reaction (PCR). Bidirectional Sanger sequencing was performed using the BigDye™ Terminator v1.1 Cycle Sequencing Kit (Applied Biosystems), following the manufacturer’s protocol. Capillary electrophoresis was carried out using the Spectrum Compact CE System (Promega). The forward primer used was 5′-CTGTCCCCTCTTTGCTGTA-3′, and the reverse primer was 5′-CCACCCAACCTTATCCTCT-3′. For the IUSM cohort the genetic testing was performed on next-generation sequencing (NGS) Primary Immunodeficiency and Lymphoid Malignancy Predisposition Panel by Prevention Genetics that included the *UBA1* gene. Genomic DNA was analyzed using targeted exome capture with the PGxome custom capture panel (Personalis, Menlo Park, CA). Sequencing was performed on a next-generation sequencing platform, and raw data were processed using the Infinity bioinformatics pipeline (version 4.3.0). The mean sequencing depth across targeted regions was 189×. The fraction of bases covered by high-quality sequencing reads was 99.6%, ensuring comprehensive coverage of the targeted exome regions.

The study was conducted in accordance with Good Clinical Practice (GCP) guidelines and the ethical principles outlined in the Declaration of Helsinki. Approval of the Institutional Review Board (IRB) was received from both IUSM and PGIMER.

### Follow-up data and statistical analysis

Overall survival (OS) was defined as the time from the date of onset of the first symptom associated with the disease to death from any cause or date of last visit. Kaplan–Meier curves were generated to estimate survival distributions. Log-rank tests were used for statistical comparisons. All statistical analyses were performed using SPSS statistical software package v29.02.

## Results

### Clinical characteristics

**Fig. 1:**
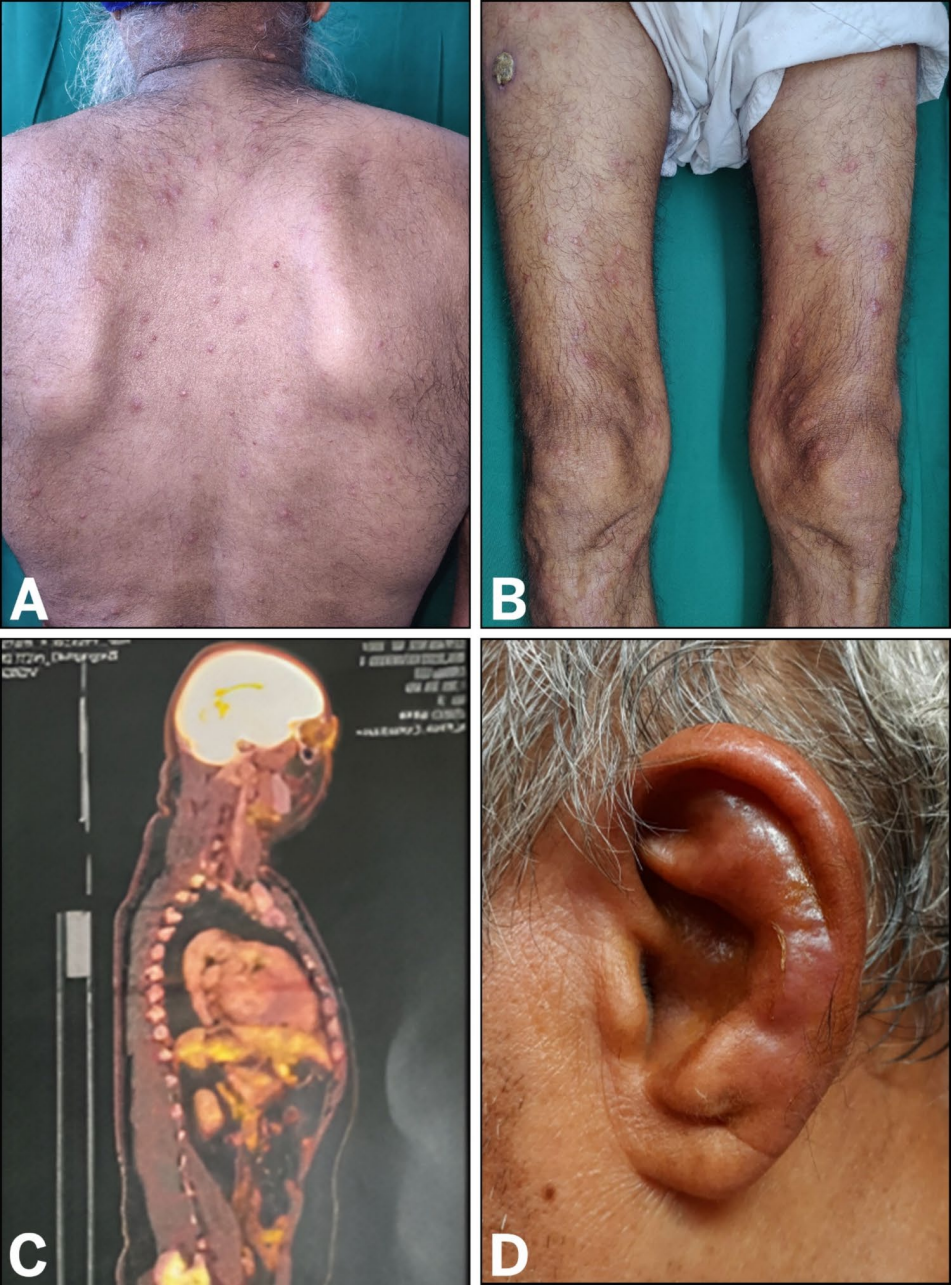
Clinical presentation and imaging. **A**, **B** Multiple discrete erythematous nodules and plaques with pseudovesiculation on the patient’s back (**A**) and thighs (**B**). **C** Positron emission tomography (PET) scan showing diffuse bone marrow uptake, indicative of increased metabolic activity. **D** Inflammation of the auricular cartilage

All the patients were male with a median age of 61.5 years (range 42–77 years). Duration of clinical symptoms ranged from 4 months to 10 years. Prominent clinical characteristics noted were a history of fever (*n* = 11), arthritis/arthralgia (*n* = 10), inflammatory skin lesions (*n* = 7), vasculitis (*n* = 6), ocular inflammatory conditions (*n* = 6), relapsing polychondritis (*n* = 6), auricular chondritis (*n* = 4) (Fig. 1), unprovoked venous thrombosis (*n* = 5). Generalized lymphadenopathy (*n* = 3) and mild splenomegaly (*n* = 2) were also present. Cutaneous lesions included erythematous rash (*n* = 4), erythema nodosum (*n* = 4), sweet’s syndrome (*n* = 4), erythema multiforme (*n* = 1) and erythematous macules (*n* = 2). Ocular inflammations included uveitis (*n* = 4) and orbital cellulitis (*n* = 1). Cutaneous small vessel vasculitis was noted in 3 of the cases, while one patient had large vessel vasculitis. Three patients had a history of unprovoked thrombosis in the form of superficial cutaneous and deep venous thrombosis in one case and only deep venous thrombosis in the other two cases. Pulmonary involvement was identified in 4 of the cases in the form of ill-defined ground glass opacities (GGOs) (*n* = 1) and lung nodules (*n* = 2). Gastrointestinal tract involvement was noted in one of the patients, presenting as acute intestinal obstruction. Two patients were diagnosed with rheumatological diseases, including systemic lupus erythematosus (SLE) and rheumatoid arthritis. Therapeutic management included glucocorticoids (*n* = 11) and conventional DMARDS (*n* = 4). Six patients responded to treatment, while five were treatment-refractory with progressive disease and ultimately died from the illness.

Clinical characteristics of the patients and treatment details are listed in Table 1. PET (Positron Emission Tomography) scan was performed in two cases which showed diffuse uptake in bone marrow (Fig. 1).

**Table 1:** Clinical characteristics, treatment details, laboratory data of patients diagnosed with VEXAS syndrome

Demographics	Cohort (*n* = 11)
Age of disease onset, years, median (range)	58 (40–75)
Age at diagnosis, years, median (range)	61 (42–77)
Gender, male, *n* (%)	11 (100)
Clinical manifestations	*n* (%)
Fever	11 (100)
Arthritis/arthralgia	10 (91)
Inflammatory skin lesions	7 (64)
Vasculitis	6 (55)
Relapsing polychondritis	6 (55)
Ocular inflammation	6 (55)
Auricular chondritis	4 (36)
Nose chondritis	1 (9)
Unprovoked venous thrombosis	5 (45)
Orchitis	0 (0)
Pulmonary infiltrates	5 (45)
Hepatosplenomegaly	1 (9)
Lymphadenopathy	3 (27)
Clinical diagnosis	*n* (%)
Sweets syndrome	4 (36)
Systemic lupus erythematosus	1 (9)
Rheumatoid arthritis	1 (9)
Stills disease/Behcet’s disease	1 (9)
Treatment details	*n* (%)
Glucocorticoids	11 (100)
DMARDs	4 (36)
Response to therapy	*n* (%)
Responding	6 (55)
Refractory	5 (45)
Death due to disease	5 (45)
**Hematological and laboratory parameters**	
Hematological and laboratory features	Cohort (*n* = 11)
**Cytopenias**	***n* (%)**
Anemia	10 (91)
Macrocytic anemia	8 (73)
Neutropenia	2 (18)
Thrombocytopenia	4 (36)
**Bone marrow findings**	***n* = 9**
Morphological dysplasia	*n* (%)
Dyserythropoiesis	5 (56)
Dysmegakaryopoiesis	4 (44)
Dysgranulopoiesis	2 (22)
Vacuolations in marrow precursors	*n* (%)
Myeloid: promyelocytes, myelocytes and metamyelocytes	4 (44)
Erythroid: proerythroblasts	1 (12)
Both	4 (44)
**Inflammatory parameters**	***n* (%)**
Raised ESR	11 (100)
Raised CRP	8 (73)
Raised IL-6	1 (9)
*UBA1* mutation analysis	*n* (%)
*UBA1* mutation (Variant c.122T>C Exon3)	6 (55)
*UBA1* mutation (Variant c.121A>G Exon3)	3 (27)
*UBA1* mutation (Variant c.121A>C Exon3)	1 (9)
*UBA1* mutation (Variant c.118G>C splice site)	1 (9)
**Additional genetic testing**	
FISH for MDS (*n* = 4)	Negative
NGS for other myeloid mutations (*n* = 3)	Negative
Karyotyping (*n* = 5)	Normal

### Hematological findings and laboratory data

**Fig. 2:**
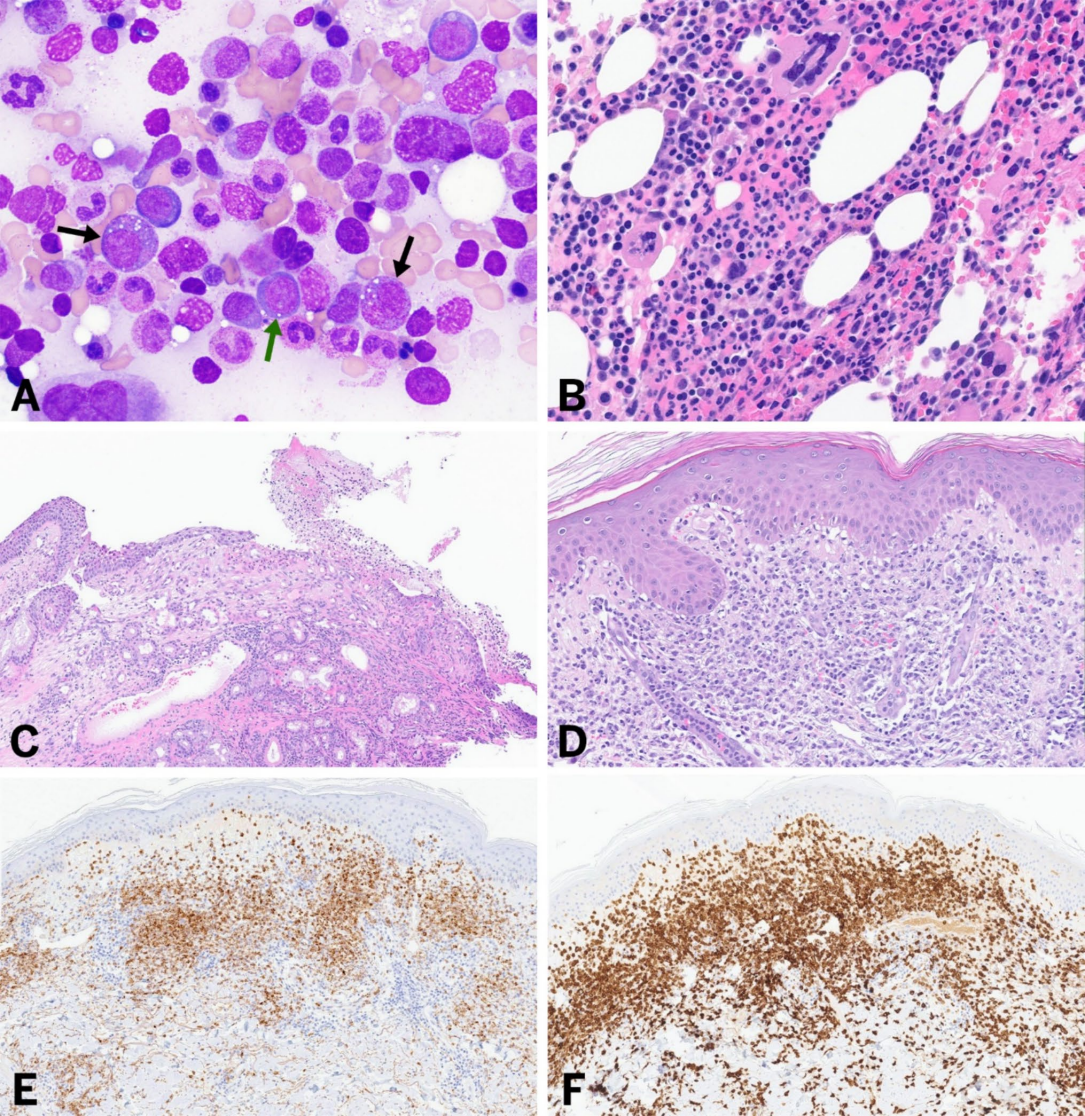
**A** Bone marrow aspirate smear (May-Grünwald stain, 100×) showing vacuolations in myelooid precursors (black arrows) and an erythroblast (green arrow). **B** Bone marrow biopsy (H&E, 40×) demonstrating megakaryocytic dysplasia including hypersegmented, hyperchromatic, and nuclear lobe separated forms. **C** Nasal septum biopsy showing acute sinusitis with abundant acute inflammatory debris. **D** Skin biopsy (H&E, 20×) showing dermal inflammatory infiltrate consistent with histiocytoid Sweet’s syndrome, comprising neutrophils, immature myeloid cells, and neutrophil karyorrhectic debris. **E** The dermal inflammatory infiltrate in skin biopsy (**D**) shows myeloperoxidase (MPO, 20×) expression, increased in mature and immature granulocytes and prominent histiocytic component expressing CD163 (20×) (**F**)

Persistent unexplained cytopenia was present across the cohort, manifesting as anemia (*n* = 10; 8 macrocytic), thrombocytopenia (*n* = 4), and neutropenia (*n* = 2). Total leucocyte counts were within normal limits (*n* = 11). Bone marrow examination were conducted in nine cases. Hypercellular marrow was observed in 7 patients, while 2 patients presented with normocellularity. An increased myeloid-to-erythroid (M:E) ratio was noted in two of them. All nine patients exhibited varying degrees of dysplasia. Dyserythropoiesis was the most frequently observed lineage dysplasia (*n* = 5, 56%), followed by dysmegakaryopoiesis (*n* = 4, 44%), and dysgranulopoiesis (*n* = 2, 22%). Significant dysplasia (> 10%) meeting WHO classification criteria for morphologic dysplasia was present in five cases. Among these, multilineage dysplasia involving two or more cell lines was identified in 4 cases (80%), while unilineage dysplasia was present in 1 case (20%). Examination of bone marrow aspirate smears revealed cytoplasmic vacuolations in hematopoietic precursor cells in all nine cases: 4 cases with vacuolations in both myeloid and erythroid precursors, 4 cases with vacuolations in myeloid precursors only, and 1 case with vacuolations in erythroid precursors only (Fig. 2). Vacuolations were also noted in monocytic lineage and eosinophils in two cases. FISH for MDS was performed in four cases; however, all were negative for 5/5q deletion, monosomy 7, trisomy 8, and 20q deletion. Two cases also had coexisting monoclonal gammopathy of undetermined significance (MGUS) characterized by kappa-restricted monoclonal plasma cells in bone marrow.

**Fig. 3:**
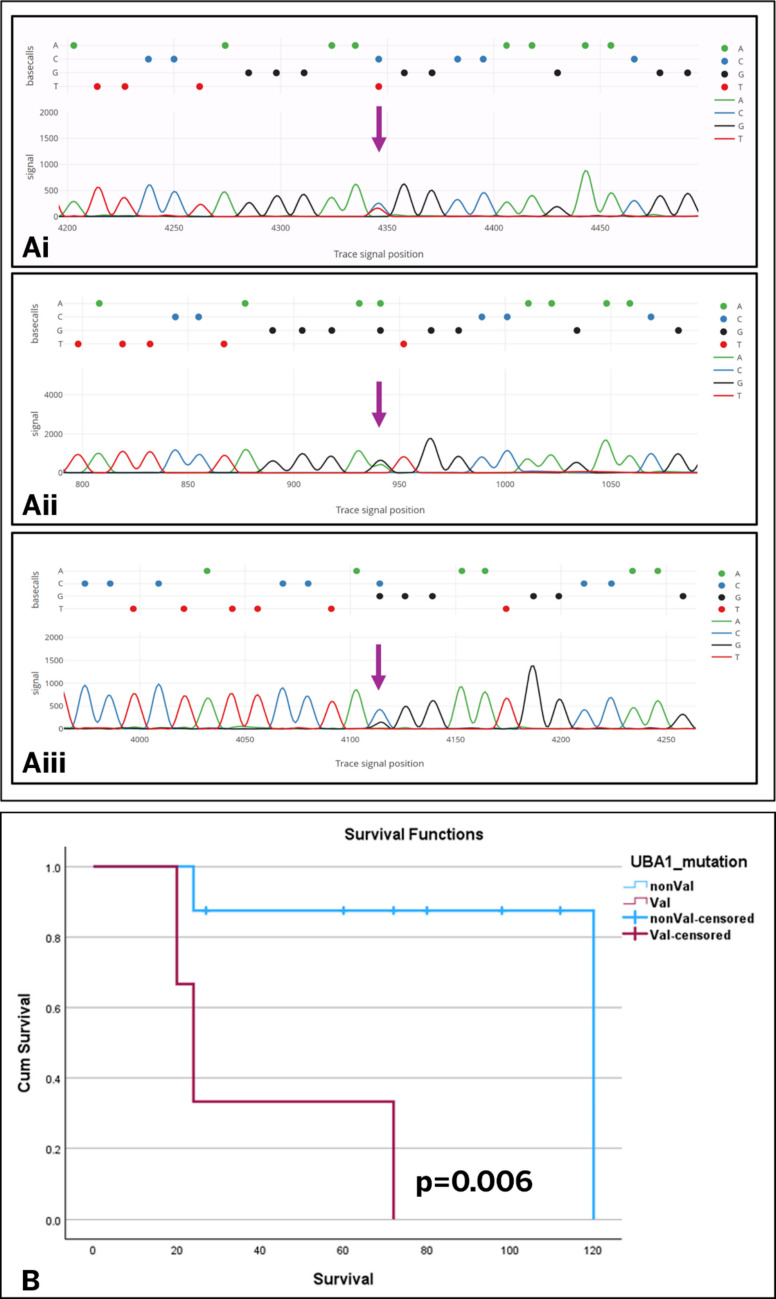
UBA1 Mutations and Patient Survival. **A** Sanger sequencing electropherograms demonstrating different UBA1 mutations: (Ai) Variant c.122T>C (p.Met41Thr) in Exon 3, (Aii) Variant c.121A>G (p.Met41Val) in Exon 3, and (Aiii) Variant c.118G>C at the splice site junction of Intron 2/Exon 3. **B** Overall survival (OS) rates of individuals stratified by their specific UBA1 genotype

*UBA1* somatic mutations included p.Met41Thr (c.122 T>C Exon 3) (*n* = 6, 55%), p.Met41Val (c.121 A>G Exon 3) (*n* = 3, 27%), p.Met41Leu (c.121A>C Exon3) (*n* = 1, 9%) and a variant in the acceptor splice site of intron 2/exon 3 boundary (c.118 G>C) (*n* = 1, 9%) (Fig. 3). Screening for additional somatic mutations associated with myeloid neoplasms by NGS was available in three patients and was negative. One patient had primary immunodeficiency and lymphoid malignancy predisposition panel heterozygous for ABCG8 c.1083G>A (p.Trp361), RAG2 c.104G>T (p.Gly35Val), and UBA1 c.121A>G (p.Met41Val), all of which are classified as pathogenic, and RBM8A c.−21G>A (pre-coding region), which is considered likely pathogenic. The Periodic Fever Syndrome panel showed no pathogenic or likely pathogenic variants in ELANE, LPIN2, MEFV, MVK, NLRP3, PSTPIP1, and TNFRSF1A. Conventional Karyotyping was performed on five patients, who showed a normal karyotype. The laboratory data, including genetic and hematological findings, are summarized in Table 1.

### Dermatopathological findings

Skin biopsies were available from four patients, all of whom showed neutrophilic dermatosis in the dermis. One patient also exhibited neutrophilic panniculitis. The typical histologic pattern consisted of papillary dermal edema and a dense superficial and deep dermal infiltrate, predominantly composed of neutrophils, along with karyorrhectic neutrophilic debris. The infiltrate was prominent in the superficial to mid-dermis and transitioned to a perivascular and periadnexal distribution in the deeper dermis. In two of these patients, admixed immature myeloid cells were identified, which were positive for myeloperoxidase (MPO), CD163, CD14, and CD33, consistent with the histiocytoid variant of neutrophilic dermatosis (Fig. 2). Interestingly, the two histiocytoid variants were associated with the p.Met41Val (c.121A>G) and p.Met41Leu (c.121A>C), respectively, while the remaining two cases harbored p.Met41Thr (c.122T>C). One of the biopsies also demonstrated perivascular neutrophils with endothelial swelling and erythrocyte extravasation consistent with low-grade leukocytoclastic vasculitis.

### Survival analysis

Follow-up of VEXAS cases ranged from 12 to 120 months, with a mean OS of 35.6 months, and 5 cases succumbed to the disease. On Kaplan- Meier analysis, p.Met41Val cases have significantly inferior OS. The mean OS was 38.6 (95% CI 5.9–71.4) months; in nonp.Met41Val cases, the OS was 108 (95% CI 76.8–139.1) months (*p* = 0.006) (Fig. 3).

## Discussion

The genotype-first approach enabled the identification of somatic UBA1 mutations as the basis of VEXAS syndrome, redefining a subset of late-onset autoinflammatory disease [1, 4, 15]. The X-linked inheritance explains the male predominance, and rising case reports since 2020 reflect increased recognition. Clinically, VEXAS exhibits marked heterogeneity, encompassing systemic inflammation, cytopenias with vacuolization and dysplasia of marrow elements, vasculitis, thromboembolism, and multi-organ involvement. Large cohorts, including the French series of 116 patients and the Italian series of seven, highlight its broad spectrum and frequent diagnostic challenges [2, 14]. Table 2 summarizes the key findings from case series published to date.

**Table 2:** Compilation of published data on VEXAS syndrome

					Rheumatological manifestations							Hematological manifestations	
Study	Cohort Size (*n*)	Genotype, *n* (%)	Age in years (range)	Gender, *n* (%)	Fever, *n* (%)	Arthritis, *n* (%)	Vasculitis, *n* (%)	Ocular findings, *n* (%)	Skin findings, *n* (%)	Pulmonary, *n* (%)	Chondritis, *n* (%)	Macrocytic anemia, *n* (%)	MDS, *n* (%)
Present study, 2025	11	pMet41Thr 6 (55) pMet41Val 3 (27) PMet41Leu 1 (9) c.118-1G>C 1 (9)	61 (42–77)	11 (100)	11 (100)	10 (91)	6 (55)	6 (55)	7 (64)	5 (45)	5 (45)	8 (73)	5 (56)
Al-Hakim et al., UK, 2025 [38]	59	pMet41Thr 25 (46) pMet41Val 12 (22) pMet41Leu 10 (19) Splice/Other 7 (13)	71 (49–79)	58 (98)	44 (75)	24 (41)	18 (31)	22 (37)	50 (85)	26 (44)	23 (39)	40 (74)	27 (46)
Beck et al., USA, 2023 [9]	11	pMet41Thr 1 (9) pMet41Val 3 (27) pMet41Leu 4 (36) Ser56Phe 2 (18) c.118-2A>G 1 (9)	65 (50–89)	11 (100)	11 (100)	7 (64)	4 (16)	2 (18)	8 (73)	10 (91)	NA	8 (73)	4 (36)
van der Made et al., Netherlands, 2022 [42]	12	pMet41Thr 12 (100)	67 (55–79)	12 (100)	11 (92)	4 (33)	2 (16)	5 (42)	10 (83)	8 (67)	6 (50)	11 (92)	6 (50)
Georgin-Lavialle et al., France, 2022 [2]	116	pMet41Thr 52 (45) pMet41Val 35 (30) pMet41Leu 21 (18) Splice 8 (7)	67 (62–77)	111 (96)	75 (65)	33 (28)	30 (26)	47 (41)	97 (84)	57 (49)	42 (36)	88 (76)	58 (50)
Muratore et al., Italy, 2022 [14]	7	p.Met41Thr	64 (46–76)	7 (100)	7 (100)	NA	7 (100)	NA	5 (71)	6 (85)	1 (14)	6 (86)	NA
Khitri et al., France, 2022 [33]	95	NA	66 (61–72)	53 (96)	33 (60)	36 (67)	3 (6)	30 (57)	44 (82)	13 (46)	52 (98)	NA	41 (75)
Ferrada et al., USA, 2022 [29]	83	p.Met41Thr 50 (60) p.Met41Val 18 (22) p.Met41Leu 15 (18)	66 (41–80)	83 (100)	69 (83)	48 (58)	NA	20 (2)	68 (82)	47 (57)	45 (54)	81 (97)	NA
Tsuchida et al., Japan, 2021 [43]	8	p.Met41Thr 3 (37.5) p.Met41Val 2 (25) p.Met41Leu 3 (37.5)	72 (66–81)	8 (100)	6 (75)	2 (25)	NA	3 (38)	7 (88)	NA	8 (100)	7 (88)	4 (50)
Bourbon et al., Italy, 2021 [32]	11	p.Met41Thr 5 (46) p.Met41Val 3 (27) p.Met41Leu 1 (9)	66 (47–83)	11 (100)	10 (91)	11 (100)	7 (64)	5 (46)	11 (100)	5 (46)	5 (46)	7 (64)	6 (54)
Koster et al., USA, 2021 [15]	9	p.Met41Thr 9 (100)	70 (65–72.5)	9 (100)	8 (88)	5 (55)	4 (44)	4 (44)	NA	NA	5 (55)	9 (100)	NA
Beck et al., USA, 2020 [1]	25	pMet41Thr 15 (60) pMet41Val 5 (20) pMet41Leu 5 (20)	64 (45–80)	25 (100)	23 (92)	NA	4 (16)	NA	2 (88)	18 (72)	16 (64)	24 (96)	6 (24)

Our study included eleven patients of VEXAS syndrome, restricted to males (100%) [2, 6, 14]. The median age at diagnosis of 61 years aligns with findings reported in most of the studies in the literature. However, young patients presenting with typical symptoms of VEXAS syndrome should be tested for UBA1 variants as Hernandez et al. have reported a case in a 23-year-old male patient [16]. VEXAS syndrome is more frequently seen in males because it is caused by somatic mutations in the X-linked UBA1 gene and males have only one X chromosome making them more susceptible to X-linked disorders. However, VEXAS can also occur in females, particularly in conditions that result in the loss of one X-chromosome, such as Turner syndrome or acquired monosomy X or mosaicism [17, 18]. Recent studies have shown that while VEXAS is much less common in women, it is nevertheless a recognized entity, with an estimated prevalence of 1 in 26,000 women over age 50 compared to 1 in 4000 men in the same age group [9, 17].

The clinical features in our cohort (Table 1) are concordant with observations by Georgin-Lavialle et al. and Beck et al., with high prevalence of fever (100%), arthritis (89%), cutaneous involvement (56%), ocular inflammation (56%), and chondritis (56%) [2, 9]. From a rheumatologist's viewpoint, VEXAS syndrome can either mimic or coexist with established rheumatologic diseases. We documented three cases of VEXAS occurring alongside one case of SLE, one of RA and one case of Behcet disease. The first reported instance of VEXAS coexisting with SLE, initially described by Sharma et al., was also included in this series [19].

VEXAS is a progressively severe disorder that presents with overlapping features of rheumatologic, hematologic, and dermatologic conditions. All patients in our cohort exhibited persistent, unexplained cytopenias, including anemia, thrombocytopenia, and neutropenia, consistent with findings reported by Beck et al. [1, 9]. Bone marrow is frequently involved in VEXAS syndrome and thus requires marrow examination. The marrow is typically hypercellular, with dysplasia affecting one or more lineages. Dyserythropoiesis and dysmegakaryopoiesis are more common. The myeloid-to-erythroid ratio may be elevated, and both myeloid and erythroid precursors frequently exhibit cytoplasmic vacuolations [3, 11, 12, 14]. These findings were reflected in our series. Seventy-eight percent (*n* = 7/9)of the cases demonstrated hypercellular bone marrow, with an increased myeloid-to-erythroid ratio observed in two cases. All patients exhibited cytoplasmic vacuolations in either myeloid and erythroid precursors, along with evidence of morphologic dysplasia. Our dysplasia frequencies (dyserythropoiesis 56%, dysmegakaryopoiesis 44%, dysgranulopoiesis 22%, with multilineage involvement in 80% of cases) are generally consistent with published VEXAS series reporting 30–50% dyserythropoiesis and dysmegakaryopoiesis, although our cohort is on the higher end for erythroid and megakaryocytic involvement [9, 12, 20]. Notably, classical cytogenetic abnormalities associated with conventional MDS, such as deletion 5q, monosomy 7, trisomy 8, and deletion 20q, as well as typical MDS-associated gene mutations in DNMT3A, TET2, and ASXL1, were absent in our cohort. This absence mirrors findings in other VEXAS series and further differentiates VEXAS-associated MDS from classical subtypes, supporting the notion of a distinct clonal entity driven predominantly by UBA1 mutations rather than the recurrent cytogenetic changes and gene mutations observed in typical MDS [12, 20]. The mutational landscape thus appears less complex, with UBA1 mutations serving as the primary driver and secondary mutations being uncommon, which lends strong support to the concept of VEXAS as a genetically distinct MDS subtype. Reflecting this evolving understanding, the WHO fifth edition of classification of hematolymphoid tumors currently lists VEXAS syndrome under the section on clonal hematopoiesis, with a note that, as further data emerge, VEXAS may ultimately be recognized as a distinct disease entity in future editions. Accordingly, we have classified the five cases with significant morphologic dysplasia as VEXAS syndrome-associated morphologically defined myelodysplastic neoplasms with low blast count. Two of our cases also exhibited hemophagocytosis; however, they did not meet the criteria for hemophagocytic lymphohistiocytosis (HLH). It is essential to be cognizant that VEXAS can trigger HLH, as reported by Grey and Kao et al. [21, 22]. UBA1 gene variants have been associated with an increased risk of myelodysplastic syndromes (MDS) and plasma cell dyscrasias, including multiple myeloma (MM) in 25–55% of cases. In our cohort, two patients were also diagnosed with monoclonal gammopathy of undetermined significance (MGUS). However, it remains unclear whether UBA1 mutations drive the development of these hematologic malignancies in the context of VEXAS syndrome [12, 23, 24].

Cutaneous involvement is highly prevalent in VEXAS syndrome, with over 80% of patients developing skin manifestations and it often happens early in the disease course [25–28]. These findings are typically seen as erythematous indurated plaques or nodules. The most common dermatopathological findings include neutrophilic dermatitis, leukocytoclastic vasculitis, leukocytoclasia, and sometimes exhibit features of neutrophilic panniculitis and chronic septal panniculitis. The histiocytoid variant of Sweet syndrome is characterized by histiocytoid cell infiltrates (immature myeloid cells) rather than classic neutrophils. It has a significant association with the p.Met41Leu variant, as demonstrated by Tan et al. [28]. In our study, two cases of the histiocytoid variant of Sweet syndrome were identified. One harbored the p.Met41Val mutation, while the other carried the p.Met41Leu mutation. Leukocytoclastic vasculitis was noted in one case.

The *UBA1* gene encodes the E1 enzyme*,* which plays a critical role in the cytoplasmic ubiquitination pathway. Acquired somatic mutations occurring at methionine-41 (p.Met41) are strongly associated with VEXAS syndrome and have been well documented in the literature [1]. The initial study by Beck et al. outlined three variants of *UBA1* mutations: p.Met41Thr (15 of 25, 60%), p.Met41Val (5 of 25, 20%), p.Met41Leu (5 of 25, 20%). Similar distributions were reported by Georgin-Lavialle et al. and Koster et al. [2, 15]. These findings were echoed in our series. Our cohort has all three common variants of the *UBA1* gene mutation: p.Met41Thr (c. 122 T>C Exon 3) (55%), p.Met41Val (c.121 A>G Exon 3) (27%), and p.Met41Leu (c.121 A>C Exon 3) (9%). Additionally, we reported a new variant in the acceptor splice site of the intron 2/exon 3 boundary (c. 118-1G>C) that affects the normal splicing necessary for translating the cytoplasmic UBA1b isoform. This mutation is believed to produce abnormal UBA1b messenger RNAs that lack the regions around p.Met41, which are essential for translating the cytoplasmic UBA1b isoform. A similar mutation was noted in a 67-year-old male patient with hematological and rheumatological symptoms in a study by Poulter et al., and it was also reported in 19% of cases in a series by Beck et al. [4, 9] Our research identified genotype-specific survival differences, revealing that p.Met41Val is associated with poorer survival than non-p.Met41Val cases. This is due to p.Met41Val supporting less non-AUG translation of the UBA1b isoform compared to other variants, suggesting that the severity of VEXAS syndrome is inversely related to the amount of residual UBA1b protein isoform, as demonstrated by Ferrada et al. [29]. Although our results are promising, the survival results are exploratory and hypothesis-generating; the limited sample size necessitates verification in a larger cohort.

VEXAS syndrome coexists with various types of vasculitis. Watanabe et al. reviewed the literature and reported all three types of vasculitis: small, medium, and large vessel vasculitis [30]. In our study, we reported four cases (44%) associated with vasculitis (three small vessel and one large vessel), similar to the findings of Koster et al. [15]. Two of our cases exhibited increased bone marrow uptake on PET scans. Bixio et al. reported PET scan findings in patients with VEXAS syndrome, noting that 77% of their cohort demonstrated increased bone marrow uptake. This pattern reflects chronic inflammation within the bone marrow microenvironment, with heightened metabolic activity often preceding clinical disease onset by 2–3 years. The degree of hypermetabolism also correlated with disease activity, as evidenced by reductions in hypermetabolic foci following therapy [31]. Additionally, pulmonary involvement was identified in 4 cases (44%), characterized by ground-glass opacities, consolidation, and radiologically detected lung nodules. Studies by Bourbon and Khitri et al. have similarly reported lung involvement in 46% of cases [32, 33].

Management of VEXAS syndrome remains challenging due to its clinical heterogeneity and frequent treatment refractoriness, necessitating individualized, multidisciplinary approaches. In our cohort, all patients received glucocorticoids, often alongside immunosuppressants such as methotrexate, mycophenolate mofetil, or hydroxychloroquine, while some were treated with biologics including anakinra and tocilizumab. Despite these multimodal regimens, therapeutic responses were variable and frequently incomplete. Notably, azacitidine has demonstrated significant efficacy in VEXAS-associated myelodysplastic syndrome, inducing sustained hematologic and molecular remissions in a substantial proportion of patients, with some maintaining long-term clinical and genetic remission even after cessation of therapy [34, 35]. Nevertheless, relapses and incomplete responses remain challenges. Allogeneic hematopoietic stem cell transplantation stands as the only established curative option but is limited by patient age and comorbidities [36–38]. Recent data suggest that while transplantation can be effective, it carries substantial risks in the typical older comorbid patients [39]. Prognosis is often determined more by inflammatory complications and burden of comorbidities than leukemic progression, highlighting the need for multidisciplinary, genetically informed care. Early diagnosis and molecular confirmation are essential for guiding therapeutic decisions and improving outcomes. Ongoing clinical trials will be vital for defining the optimal timing of transplantation and refining personalized treatment strategies for this complex syndrome.

Consistent with prior reports, VEXAS syndrome is a severe and progressive disorder. In our series, five patients (45%) experienced refractory disease and died within three years of diagnosis. Complete cause-of-death data are unavailable due to the retrospective nature of our cohort; however, many patients originated from rural and remote areas with limited access to subspecialty care, and several were diagnosed only after a prolonged disease course. Available clinical information suggests the high mortality reflects delayed diagnosis, untreated disease, refractory inflammation, and disease-related complications rather than treatment toxicity. These findings highlight the critical importance of early recognition and timely treatment to optimize outcomes.

Finally, our study has limitations, including a small sample size, retrospective, multi-institutional composition with variable availability of advanced testing, incomplete disease specific mortality documentation, and a relatively short follow-up period which are reflective of the recent identification of this syndrome and the challenges inherent in studying emerging diseases. Consequently, our results are exploratory and require validation in larger prospective cohorts with standardized protocols. However, our analysis contributes to clinicopathologic characterization from an underrepresented geographic region.

## Conclusion

In conclusion, VEXAS syndrome should be considered in older male patients presenting with refractory autoimmune manifestations, including recurrent fevers, arthritis, vasculitis, cutaneous lesions, chondritis, and pulmonary infiltrates, particularly when accompanied by hematologic abnormalities such as macrocytic anemia, thrombocytopenia, myelodysplasia, and vacuolation of myeloid and erythroid precursors. This case series represents the first comprehensive cohort-based and systematic clinicopathologic and genetic characterization of VEXAS syndrome from India, supplemented by two additional patients from the United States. Although isolated Indian cases have previously been described, our study provides an in-depth analysis of nine patients and offers valuable insights into the diverse clinical and laboratory spectrum of this recently defined disorder using multi-institutional referral data [7, 40, 41]. Early and accurate recognition remains essential given the substantial morbidity and mortality associated with VEXAS syndrome.

## Data Availability

No datasets were generated or analyzed during the current study.
